# The Role of Providers and Influencers in the Use of Social Media as Solace for Psoriasis: Qualitative and Quantitative Study

**DOI:** 10.2196/29904

**Published:** 2021-07-28

**Authors:** Sarin H Pakhdikian, Benjamin K P Woo

**Affiliations:** 1 Department of Psychiatry Olive View Medical Center University of California, Los Angeles Sylmar, CA United States; 2 College of Osteopathic Medicine of the Pacific Western University of Health Sciences Pomona, CA United States

**Keywords:** psoriasis, psychodermatology, social media, Instagram, coping skills, stigma, depression, anxiety, psychosocial, influencers, internet

## Abstract

**Background:**

Psoriasis is a multisystem chronic inflammatory skin disease and is a relatively common disorder in children and adults. The burden of psoriasis impacts both the physiological and psychological areas of one’s life. Given the robust use of the internet and social media, patients have turned to Instagram for educational and social support to discuss psoriasis.

**Objective:**

This study aimed to characterize how patients interact with Instagram to cope with the biopsychosocial aspects of psoriasis. We analyzed journals and organizations, and compared them with the public profiles of individuals diagnosed with psoriasis who provided information and refuge. Our goal was to identify how followers engaged and what type of content they were most receptive to in terms of psoriasis.

**Methods:**

All journals and organizations representing psoriasis were selected for review. The top 10 public profiles of individuals diagnosed with psoriasis were also selected for comparison. The numbers of followers, followings, and posts were noted to evaluate popularity. The numbers of likes and comments were also recorded to understand engagement.

**Results:**

On comparing journals and organizations to public profiles, we found that the former had a greater number of followers but engaged less with the audience on Instagram based on the number of profiles they followed. Profiles of individuals with psoriasis produced content that was more personal and relatable, including experiences with flares, motivational text, and emotional support. The content produced by journals and organizations was geared toward education and providing peer-reviewed resources and commentary from licensed health care professionals. Followers were more engaged via “likes” than “comments” on the Instagram profiles of journals and organizations, as well as the public profiles of individuals diagnosed with psoriasis.

**Conclusions:**

There was evident online presence of journals and organizations, and public profiles of individuals providing content regarding psoriasis on Instagram. However, there were distinguishing features for the type of content being produced. Journals and organizations took the traditional approach in providing evidence-based information, whereas the public profiles of individuals provided content related to the psychosocial needs of the psoriasis community. The 10 profiles of individuals provided posts involving creativity and real experiences, which were evidently well-received based on “likes” and “comments.” This research helps us appreciate what the audience on Instagram is looking for to further address how we can merge these needs to provide a holistic platform on Instagram for both providers and patients. Social media creates a space for collaboration, which can be advantageous for journals and organizations to work with patient volunteers from diverse backgrounds who can help build a therapeutic alliance and public presence on Instagram with their viewers in order to deliver medical peer-reviewed information.

## Introduction

Psoriasis is a multisystem chronic inflammatory skin disease and is a relatively common disorder in children and adults. There are many risk factors associated with the disease, including genetic, environmental, and behavioral factors. The primary pathophysiology of psoriasis is mediated by the immune system, where T lymphocytes, dendritic cells, and cytokines play important roles [1]. Patients typically present with scaling, induration, and erythema of the skin, which leads to hyperproliferation and abnormal differentiation of the epidermis, vascular dilatation, and inflammatory cell infiltrates [2].

The burden of psoriasis impacts both the physiological and psychological areas of one’s life. The prevalence of depression and anxiety in patients with psoriasis is significantly higher than that in the general population [3]. On comparing quality of life between those with psoriasis and those with different dermatological conditions, discrepancies have been observed. Patients diagnosed with psoriasis have been compared to those with urticaria, acne, nonatopic eczema, and alopecia [4]. The findings showed that those with psoriasis most often reported stress as the predominant factor in disease exacerbation, which impacted their activities of daily living more frequently than other conditions. Evidence based on systematic reviews of the literature on the psychopathology in psoriasis has indicated that stress is the driving factor of onset, exacerbation, and relapse [5]. The economic impact of psoriasis has been studied by examining the costs of various therapeutic options. The average monthly expense for treatment ranges from US $100 to $200, and this is prior to the consideration of the severity of the disease [6]. The financial burden of this disease is associated with lower quality of life as severity increases, which can further exacerbate the psychosocial stressors. Psychodermatology addresses the interaction between the mind and the skin. While psychiatry is focused on the internal nonvisible disease, dermatology explores the external visible aspects. A neuroimmunocutaneous system has been described as the interplay between these two specialties, where the courses of inflammatory skin diseases and psychiatric conditions disrupt this inherent system [7]. Visibility of lesions significantly affects body image in this patient population, often leading to stigmatization, and it has negative effects on psychological health.

Our interest lies in how patients cope with the psychological impacts of the illness using social media, specifically Instagram, as a resource. The increase in internet users in the past few decades has been exceptional, with 8% activity in 2005 rising to 74% in 2012 [8]. Various social media platforms are readily available for discussion on everyday trials and tribulations, including dermatology. Given the visual component of scrolling through a media feed, platforms like Instagram can be a cornerstone in providing education and resources for patients with psoriasis. Previous research has explored Facebook, Twitter, YouTube, and LinkedIn as means of communication [8-11], but limitations consistently point toward the lack of medical resources presented in a way to garner enough attention of users and the targeted audience [9].

Given the robust use of the internet and social media, we explored how journals and organizations, as well as public accounts on psoriasis, discuss education or personal experience in the context of their posts on Instagram. We studied how this information is received and which profiles had the most popularity (or engagement) based on their social media presence. We further investigated the possible implications of the utility of these applications for communication between health care professionals and patients.

## Methods

### Data Collection

We began collecting data by searching Instagram for notable journals and organizations that study and educate the public about psoriasis. The first 50 public posts of each account were studied for performance based on the number of followers, the number of accounts followed, and audience interaction through likes and comments.

Four Instagram accounts with respect to journals and organizations specific to psoriasis included the *Journal of American Academy of Dermatology*, *Journal of the American Medical Association* (*JAMA*) *Dermatology*, American Academy of Dermatology Association, and National Psoriasis Foundation. Psoriasis Speaks and the *Journal of Psoriasis and Psoriatic Arthritis* did not have an Instagram presence.

It is important to clarify that although *JAMA* *Dermatology* had a direct link to their Instagram account on their webpage, the Instagram account itself encompassed the entire JAMA Network, reflecting all medical specialties. It was included in our research for completeness. We surveyed the posts for all material related to dermatology and then filtered specifically for psoriasis.

Next, public Instagram accounts of individuals who are advocates for psoriasis were reviewed. There were more than a dozen accounts that represented people living with psoriasis; however, we limited our search to profiles that explicitly included “psoriasis” or “skin” (or other terms related or implying psoriasis, such as spot, spotty, and spotted) in their Instagram handle. The first 10 accounts with a prominent social media presence were reviewed. These were @getyourskinout, @psoriasis_thoughts, @beautifullyspotted, @overcoming_psoriasis, @fixmypsoriasis, @klmpsoriasis, @spottiettoohottie, @cyapsoriasis, @thegirlwithpsoriasis, and @pspotted.

### Data Analysis

This is both a qualitative and quantitative study. Quantitatively, we gathered statistics based on the numbers of followers, followings, and posts by each account in order to understand the popularity of these profiles. Next, we studied the data on the numbers of likes and comments to interpret receptivity from the Instagram community. Qualitatively, we observed the context of the posts on each account, limited to the first 50 as previously mentioned, to explore which ideas and experiences are well received based on the numbers of likes and comments on each post.

By summarizing the numbers of followers, followings, and posts, we were also able to assess the visibility of the content. More likes and comments increase online presence, and therefore given the algorithms of social networks, they increase the visibility of the profiles themselves.

In our qualitative analysis, the themes we were interested in exploring were who, what, and how people responded to the content on Instagram. To identify the “who,” we observed if the content was produced by physicians or people with psoriasis. To answer the “what,” we explored the type of content being produced, for example, educational versus personal experiences with flares, treatment, and psychosocial aspects of psoriasis. To investigate the “how,” we inquired if the audience engaged more so by liking the post or commenting on it.

We excluded video posts on Instagram from our data analysis as this feature was not utilized on the platforms of journals and organizations during the timeframe of our search. We will comment on the use of videos briefly in the Discussion section.

The described work presents minimal risk research. We used public user data from Instagram, adhering to the terms and conditions, terms of use, and privacy policies of Instagram. Any identifying and personal health information was redacted from the profiles.

## Results

The data presented in the tables below were reflective statistics as on April 8, 2021.

From the six major journals and organizations that represent psoriasis and speak about psoriasis, only four had a presence on Instagram. The opposite was true when searching for public profiles of individuals representing or advocating for psoriasis. Therefore, we had to limit our consideration of public profiles to the top 10 accounts that personified psoriasis through their content. We did not review profiles that had personal names in their account screen names.

Table 1 presents the journals and national organizations on Instagram organized by the number of followers, number of people they followed, and number of posts. The JAMA Network profile, as discussed above, was included for completeness after filtering for dermatology and then psoriasis in particular.

**Table 1:** Instagram profile data of medical journals and national organizations.

Name	Followers, n	Followings, n	Posts, n
JAMA Network	84,900	82	423
*Journal of the American Academy of Dermatology*	44,700	291	634
American Academy of Dermatology	63,100	452	802
National Psoriasis Foundation	18,000	327	852

The results showed that journals and national organizations had a larger group of followers but had a relatively low number of accounts they interacted with, indicated by their “followings.” The mean number of posts by journals and national organizations collectively was 667.8 (SD 193.7). Within the context of these posts, summarized in Table 2, the American Academy of Dermatology failed to produce any content on psoriasis. The JAMA Network had a total of 432 medically related posts, and there were only 20 posts that focused on dermatology and only one that discussed psoriasis. The post illustrated the inflammatory process in psoriasis. The text provided a link to a JAMA article for more information on the topic. The *Journal of the American Academy of Dermatology* produced two posts related to medical complications secondary to psoriasis, as well as alternative medicine as a therapeutic option. The National Psoriasis Foundation produced 43 posts related to psoriasis, and its impact on other organ systems, as well as one’s personal and professional life. There were various infographics of patient testimonies that provided feedback and support to the online psoriasis community. Treatment options were also discussed in their content. It was observed that for the mentioned accounts, the audience participated to a greater extent via “likes” than “comments.” On average, there were collectively 805.5 (SD 361.5) likes compared to 16.0 (SD 7.3) comments. The people who provided the information were cited as physicians or postdoctoral scholars. Each post included a peer-reviewed source that the audience could read if they needed more information that was not provided. The illustrations were consistent throughout multiple posts, using the same color scheme and inclusion of relevant logos or trademarks. There was no content created by patients or other individuals dealing or working with psoriasis. In addition, there were no pharmaceutical advertisements targeted at the treatment of psoriasis. Therapeutic options were explained by physicians. In summary, the popularity of these posts was driven by “likes.”

**Table 2:** Categorization of identified posts and audience engagement based on journals and national organizations.

Name	Number of related posts^a^	Summary of content (“What”)	Information provider (“Who”)	Number of likes (“How”), mean or mean (SD)	Number of comments (“How”), mean or mean (SD)
JAMA Network	1	Pathophysiology of psoriasis	Physician	839	9
*Journal of the American Academy of Dermatology*	2	Psoriasis-related medical issues and alternative medicine as a treatment	Physicians and researchers	1149 (553)	15.5 (11.5)
National Psoriasis Foundation	43	Psoriasis and the impact in other organ systems, personal and professional life, patient testimonies, and treatment options	Physicians, researchers, patients, and unspecified content creators	438.4 (619.3)	23.6 (32.7)
American Academy of Dermatology	0	N/A^b^	N/A	N/A	N/A

^a^The number of related posts is based on the first 50 recent posts on the social media page.

^b^N/A: not applicable.

Table 3 outlines the top 10 public profiles reviewed that were related to psoriasis. The profile data were noted similarly to the journals and national organizations. Although, on average, they had a lower number of followers, these profiles engaged more with the Instagram network as shown by the increase in people the accounts themselves followed. The relationship between followers and followings appeared to be more evenly distributed. Collectively, the 10 public profiles had a mean of 264.7 posts (SD 217), which was less compared with that of journals and national organizations.

**Table 3:** Instagram profile data on public profiles advocating for psoriasis.

Name	Followers, n	Followings, n	Posts, n
@psoriasis_thoughts	15,100	1309	361
@cyapsoriasis	11,600	3548	182
@getyourskinout	10,200	1711	652
@fixmypsoriasis	3940	813	141
@beautifullyspotted	3719	1320	162
@overcoming_psoriasis	3547	7141	594
@thegirlwithpsoriasis	2628	302	28
@pspotted	2067	791	150
@klmpsoriasis	1549	664	335
@spottiettoohottie	1465	1422	42

With regard to these public profiles on Instagram advocating for psoriasis, each one presented a quantifiable number of posts about the topic. The content of these profiles included personal experiences and struggles of psoriasis, most commonly speaking about flares and how they influenced their mood, behaviors, and self-esteem, as summarized in Table 4. Popularity was not based on how many posts these accounts had, but resonated more so with the type of content that was posted. @getyourskinout produced content that spoke about psoriasis 100% of the time, but had less popularity when compared to other accounts based on “likes” and “comments.” The Instagram account that had the highest average number of likes was @psoriasis_thoughts, which created content to promote psoriasis webinars, poetry to reflect personal experiences with psoriasis, and photo updates of flares. This account also had the largest number of followers. The account with the least number of likes was @overcoming_psoriasis, which created content that solely focused on treatment. This account was selling its own brand of products targeted for psoriasis treatment. @pspotted used the Instagram platform to talk about clinical trials available for psoriasis interventions. The posts were not advertisements, but rather a journey of the progress through treatment. From the 10 accounts reviewed, it was the only one that educated the audience on variable options for care. The account, however, was not run or managed by a licensed health care professional.

**Table 4:** Categorization of identified posts and audience engagement based on public profiles advocating for psoriasis.

Name	Number of related posts^a^	Summary of content (“What”)	Information provider (“Who”)	Number of likes (“How”), mean (SD)	Number of comments (“How”), mean (SD)
@psoriasis_thoughts	31	Psoriasis webinars, poetry, and personal experiences about flares	Individual with psoriasis	1127.3 (511.2)	121.3 (81.4)
@cyapsoriasis	21	Sponsored treatment and personal experiences about flares	Individual with psoriasis	285.1 (248.3)	17.5 (15.8)
@getyourskinout	50	Personal experience and reflection living with psoriasis and its contributions to all aspects of one’s life	Individual with psoriasis	491.4 (256.3)	20.1 (15.7)
@fixmypsoriasis	40	Treatment options, and personal experiences during pregnancy and flares	Individual with psoriasis	147.1 (49.7)	20.9 (16.4)
@beautifullyspotted	14	Motivational text and personal experiences about flares	Individual with psoriasis	163.0 (85.1)	19.7 (12.1)
@overcoming_psoriasis	35	Selling products, personal testimonies, and experiences	Individual with psoriasis	53.0 (32.9)	5.5 (7.3)
@thegirlwithpsoriasis	24^b^	Selfies with motivational text regarding psoriasis and flare updates	Individual with psoriasis	303.2 (195.2)	37.3 (30.1)
@pspotted	20	Clinical trial journey, motivation, and self-care	Individual with psoriasis	229.7 (150.6)	16.9 (16.6)
@klmpsoriasis	11	Makeup and fashion tips to boost confidence in people dealing with psoriasis	Individual with psoriasis	119.3 (46.9)	151.0 (12.4)
@spottiettoohottie	4^c^	Raised awareness about psoriasis	Individual with psoriasis	557.8 (248.1)	61.5 (43.6)

^a^The number of related posts is based on the first 50 recent posts on the social media page.

^b^This account only had 28 posts in total; videos were excluded from the count.

^c^This account only had 42 posts in total; videos were excluded from the count.

On average, followers engaged more so via “likes” (mean 347.7, SD 317.2) than “comments” (mean 47.2, SD 49.8), as observed similarly in the journal and national organization profiles. The exception was @klmpsoriasis, with followers, on average, engaging more via comments. The content was based on makeup and fashion tips to boost confidence in people dealing with psoriasis and its impact on their self-esteem. It was the only account that strayed away from educational or reflective content and was geared heavily on the psychosocial factors of psoriasis. @beautifullyspotted provided various motivational images and text to influence followers toward a mindful approach of dealing with psoriasis as a chronic condition. @cyapsoriasis had a lot of sponsored content on the Instagram page to treat psoriasis. The products that were being showcased were also used to describe personal experiences about flares and their resolution secondary to the use of these treatments.

@fixmypsoriasis depicted psoriasis in conjunction with pregnancy. Each flare throughout the course of a 9-month pregnancy was discussed and reflected upon. The content creator briefly touched on heritable concerns of psoriasis, as well as fatigue secondary to flares, and childbirth and childcare itself. @thegirlwithpsoriasis was the most “traditional” Instagram account, as it was filled with “selfies” accompanied by motivational texts, which were geared toward self-care. @spottiettoohottie had the least number of psoriasis-related posts, but was the second highest account in terms of user popularity based on the average number of likes. The content on psoriasis was geared toward raising awareness about the condition.

## Discussion

### Principal Findings

Psoriasis is a prevalent skin condition that impacts many people’s lives across the world [12]. The internet is a staple that connects over a billion people with one another. Its use in educating, connecting, and advocating for those with psoriasis is instrumental. As many more health care professionals begin to appreciate its role in medicine, a sense of community can be built with it. Technology has created a platform to address the learners’ needs (in our discussion, the patients’ needs). The concept of “digital natives” explores the usability and convenience in receiving educational information online [13]. There is evidence of an increase in the number of patients who seek medical advice from the internet [14,15]; therefore, all medical specialties should become more comfortable navigating various social media platforms to become a part of the conversation online.

There are six prominent national journals and organizations that pertain to psoriasis. Based on their social media presence only four are engaged on Instagram (*Journal of the American Academy of Dermatology*, *JAMA* *Dermatology*, American Academy of Dermatology Association, and National Psoriasis Foundation). *JAMA Dermatology* was collectively a part of the JAMA Network Instagram account. Psoriasis Speaks and the *Journal of Psoriasis and Psoriatic Arthritis* did not have an Instagram presence.

Based on the above results, the majority of the content these organizational Instagram profiles provided spoke about the pathophysiology and therapeutic options for psoriasis. The content was cited by peer-reviewed sources and detailed the names of the health care professionals who were providing the information. Consumers of online health information are generally skeptical about misinformation [16,17]. Therefore, the approach of providing resources and citations is the transparency that many patients expect from their providers, which is a reputable way to build partnership with the online community [17,18]. What makes social media, and Instagram in our discussion, unique is the range of formality this network can take. One can take time to design and publish information in a thoughtful way or engage with a natural spontaneous post, more in line with the “real-time” connection social media promotes [19]. The use of such networks has been shown to improve the patient-provider relationship, where patients felt empowered to assert their decision-making skills based on information provided on platforms that were accessed on a daily basis [20]. Only one profile, the National Psoriasis Foundation, incorporated real patients with real experiences. It is important to understand the way content is consumed by people on social media, who are evidently the drivers of its popularity. Instagram is perhaps the most visual platform, which allows posts, videos, and real-time interactions [15,21]. Its relative ease of use allows many people to engage and connect. Given that the National Psoriasis Foundation focuses solely on psoriasis, it would likely be a more frequently accessed page by followers looking for organizations related to psoriasis on Instagram.

The receptiveness and popularity of the content created by the top 10 public profiles were the “real experiences,” which captivated a larger audience based on the data from “likes” and “comments.” Their content tended to be more personal and relatable to the average viewer, but it failed to include educational material at the level of peer-reviewed sources or health care professional citations. It is important to recognize the value of evidence-based medicine, which promotes preventative and therapeutic care. Influencers on social media may blur the lines on what is in fact evidence-based versus content that is driven by sponsorship from pharmaceutical companies or other products related to psoriasis. Although some of these profiles shared brand name products used during psoriasis flares, it was unclear whether they were sponsored by these companies, presenting the risk of conflicts of interest. It would be recommended that moving forward, influencers strive for greater transparency in the driving factors of their content, with appropriate reconciliation from medical professionals. What the public profiles of individuals with psoriasis do appreciate, however, is the psychosocial implications psoriasis has on one’s mental health, self-efficacy, and self-esteem.

It is important to point out that the journals and organizations had a higher number of “likes” and “comments,” which may help us differentiate why each account is popular to begin with. The journals and organizations serve the audiences’ need for reputable resources, while the individual profiles allow followers to relate to the whole person impacted by psoriasis. This research helps us appreciate what the audience on Instagram is looking for, but the question lies in how we merge these needs to provide a more holistic platform on Instagram for both providers and patients, while avoiding the dangers of misinformation that is readily found online.

The posts curated by the individuals with psoriasis aim to reduce stigma and bias by forming an online community of “psoriasis warriors.” What the journals and national organizations fail to portray on their Instagram accounts is made up for by the individuals who share vulnerable experiences of flares, treatments, and psychologic effects of psoriasis on their personal public accounts. Given the growth surrounding psychodermatology, it is important to consider how engaging individuals with specific dermatologic diseases and their related internal nonvisible diseases via platforms, such as Instagram, should be used more readily to provide a source of information and support. Social media provides the opportunity to build and establish a reputable online presence. Conversations online are not necessarily private; thus, it is important to recognize both the positive and negative feedback that comes along with it [22], such as the boundary of professional versus personal advice. The use of social media is also beneficial to those having limited access to medical care, who can turn to these networks for evidence-based medical advice [23]. Therefore, it is important for medical professionals to gain an online presence to provide information that is free from bias to navigate patients to accurate medical information for both physiological and psychological purposes. In addition, it creates a platform for collaboration, which can be advantageous for journals and organizations to work with patient volunteers from diverse backgrounds who can help medical professionals gain a therapeutic alliance and public presence on Instagram with their followers in order to deliver evidence-based medical information.

### Limitations and Future Directions

We acknowledge that our study was limited to exploring only Instagram under the context of psychodermatology. To further strengthen the validity of our study, future research can incorporate and cross-analyze the way in which people may respond differently on Facebook, Twitter, and Instagram, as well as other related social networks. In general, likes are more amenable to gauge receptiveness to content, while comments allow for a greater understanding of how and what people may take away from the post. Future studies can dissect the comments under these posts to discuss the types of conversations prompted by content on social media.

Another limitation of our study was that not all public profiles of individuals advocating for psoriasis on Instagram were reviewed, as compared to all the journals and organizations that we included. Our methods involving screening for the top 10 profiles were limited to direct text that involved “psoriasis” or its related descriptors “spotted” and “spottie.” To allow for a more complete review, all public profiles should be taken into consideration. In between large organizations and individual content creators, there are dermatologists with large Instagram followings. It is important to consider how they engage and provide content on psoriasis to their audience.

The video function is a relatively new feature on Instagram. We did not include videos in our analysis in order to keep our discussion focused on imagery that was photo based. This limits our ability to assess if videos would be a noteworthy function to create a more personal experience between health care providers and followers online.

Further recommendations include surveying the validity of the content produced online by nonmedical professionals. It is important to observe how readily the information provided is backed by evidence-based research or consulted with medical professionals. Therefore, once medical professionals develop a more consistent presence on their respective media platforms, we can revisit the effectiveness in the delivery of information, as well as the reception by followers online.

## Abbreviations

JAMA: Journal of the American Medical Association
